# Targeting Carbohydrates and Polyphenols for a Healthy Microbiome and Healthy Weight

**DOI:** 10.1007/s13668-019-00281-5

**Published:** 2019-06-03

**Authors:** Matthias Van Hul, Patrice D. Cani

**Affiliations:** Université catholique de Louvain, WELBIO- Walloon Excellence in Life Sciences and BIOtechnology, Louvain Drug Research Institute, Metabolism and Nutrition Research Group, UCLouvain, Av. E. Mounier, 73 box B1.73.11, B-1200 Brussels, Belgium

**Keywords:** Obesity, Gut microbiota, Polyphenols, Dietary fibers, Prebiotics

## Abstract

**Purpose of Review:**

In this review, we focus on microbiota modulation using non-digestible carbohydrate and polyphenols (i.e., prebiotics) that have the potential to modulate body weight.

**Recent Findings:**

Prebiotics derived from plants have gained the interest of public and scientific communities as they may prevent diseases and help maintain health.

**Summary:**

Maintaining a healthy body weight is key to reducing the risk of developing chronic metabolic complications. However, the prevalence of obesity has increased to pandemic proportions and is now ranked globally in the top five risk factors for death. While diet and behavioral modification programs aiming to reduce weight gain and promote weight loss are effective in the short term, they remain insufficient over the long haul as compliance is often low and weight regain is very common. As a result, novel dietary strategies targeting the gut microbiota have been successful in decreasing obesity and metabolic disorders via different molecular mechanisms.

## Introduction

The prevalence of obesity continues to increase dramatically worldwide [1] and is considered a major threat to public health [2]. Obesity is associated with low-grade chronic inflammation, which contributes directly to detrimental health consequences such as metabolic disorders including cardiovascular diseases, liver steatosis, insulin resistance, and type 2 diabetes mellitus [3, 4]. The precise etiology of this obesity-related proinflammatory status remains to be fully elucidated, but dysfunction of the adipose tissue has long been regarded as the main culprit [5]. Today, it is generally accepted that changes in gut homeostasis could represent another potential source of inflammation. Indeed, over the last few years, the gut microbiome has been implicated in both beneficial and negative health outcomes in humans (for review [6]). The gut microbiota refers to the microbial community that resides in our intestinal tract. These inhabitants of the human body are separated in different phyla. Among them, the phyla Firmicutes, Bacteroidetes, and Actinobacteria represent approximately 90% of the microbiota, with the remaining portion belonging to the phyla Proteobacteria and Verrucomicrobia.

In 2006, a series of seminal papers led by Prof. Jeff Gordon and colleagues were the first to demonstrate a difference in the composition of the gut microbiota (i.e., a shift in the Firmicutes to Bacteroidetes ratio) and in the quantity of specific microbial metabolites such as short-chain fatty acids (SCFAs) between obese and lean humans or rodents [7, 8]. Following these findings, different studies encouraged a better in-depth analysis of the microbiota at the taxonomical level (i.e., family, genus, and species). More recently, disturbances in diversity and composition of the gut microbiota have been associated with a wide variety of inflammation-related disorders and pathologies including metabolic disorders such as obesity and diabetes [7, 9], inflammatory bowel disease [10, 11], autoimmune diseases [12], allergy [13], and neurological disorders [14].

It is now becoming more and more clear that there exist many host-microbe interactions, mediated through the release of bioactive molecules by bacteria in the gut and absorption of these metabolites into the circulation. Two key groups of such metabolites are SCFA, from the fermentation of non-digestible carbohydrates, and the by-products of polyphenols.

### Carbohydrates

#### Carbohydrates, Fibers, and Health: A Brief History

The impact of dietary fibers on health has been described over the last 40 years. Already in 1975, Trowell suggested that the etiology of type 2 diabetes could come from changes in the dietary habits and the lack of dietary fibers in the diet. It was also mentioned that the potential effect of sudden changes in dietary habits could partly explain the very high prevalence of diabetes observed in the Pima Indians. These hunter-gatherers shifted their classical diet—highly rich in non-digestible carbohydrates (i.e., fibers)—to a Western diet very low in fibers and cereal products [15]. In 1976, David Jenkins, another pioneer in this field, published a paper showing that the addition of non-digestible carbohydrates to the diet (i.e., guar and pectin fibers) “decreased markedly the rise in blood-glucose between 30 and 90 minutes also resulted in significantly lower insulin levels between 30 and 120 minutes” postprandially [16]. In these studies, the initial mechanism proposed was the “gel-forming” effect and the potential viscosity of the non-digestible carbohydrates. Indeed, pectin or guar gum may contribute to the so-called bulking effect (i.e., water retention), which was assumed to be the major mechanism explaining the effects observed [16–18]. Today, the positive association between non-digestible carbohydrates and health is undeniable and has been confirmed many times; however, it appears that their mechanisms of actions are more complex and cannot be explained merely by their bulking capacity.

#### Non-digestible Carbohydrates and Microbiota-Derived Metabolites

With growing insight into the role of non-digestible carbohydrates, it has become clear that their fate in the gastrointestinal tract is quite different than that of digestible carbohydrates, which are fully absorbed. Indeed, non-digestible carbohydrates escape the digestion that occurs in the first part of the gastrointestinal tract and find their way to the ileum and the colon, where they are digested by the gut microbes residing there.

**Fig. 1:**
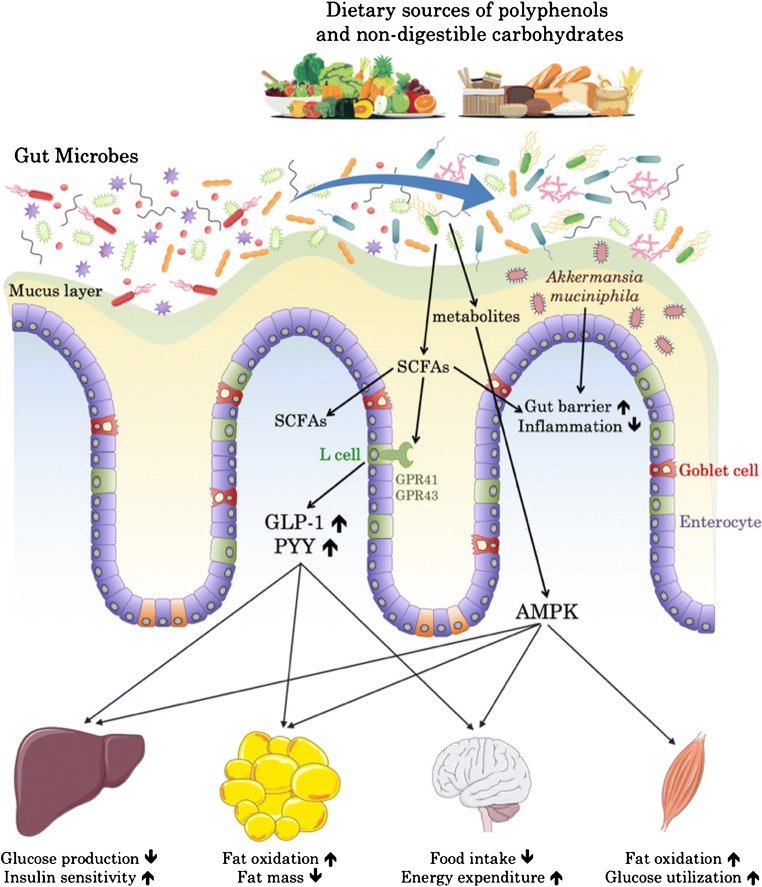
Non-digestible carbohydrates and polyphenols present in food can induce a shift in the microbial composition and favor beneficial bacteria like *Akkermansia muciniphila*. In addition, they can be fermented into short-chain fatty acids (SCFAs) or other metabolites that can either act locally, on epithelial cells and their receptors, or enter the blood circulation. SCFAs can activate receptors GPR41/43 and thereby increase GLP-1 and PYY hormone levels, while metabolites from polyphenols are able to activate AMPK in a broad range of tissues. Ultimately, both pathways result in the activation of cellular programs that favor the release of energy and promote energy expenditure

This fermentation of carbohydrates in the lower parts of the gut is associated with the proliferation or the reduction of different bacteria but also with the production of several end products such as the SCFAs (e.g., acetate, butyrate and propionate) (Fig. 1). Although SCFAs have been studied in various situations and linked with gut health (for review [19–21]), it is worth nothing that those compounds can also reach the circulation and spread to different peripheric organs (e.g., the liver, the adipose tissue, the brain [22, 23]). As a result, they have been implicated in the regulation of energy, glucose, and lipid homeostasis and they have also been closely associated with the regulation of immunity and cancer [19]. Thus, these microbial metabolites have abundant physiological effects.

#### Non-digestible Carbohydrates, Microbes, and Body Weight Regulation

The mechanisms explaining how fermented carbohydrate fibers improve metabolism have been initially revealed in rodents by using inulin-type fructans as prebiotics (e.g., inulin or oligofructose). We and others have contributed to the elucidation of the molecular mechanisms explaining how prebiotic feeding is associated with a decrease in body weight, fat mass gain, insulin resistance, and reduced food intake in rodents with genetic or diet-induced obesity [24–28]. Based on the reasoning of a potential impact of the microbial fermentation on a gut to brain axis, we discovered that fermentation of prebiotics leads to the modulation of gut peptides produced by the L-cells such as glucagon-like peptide-1 (GLP-1) and peptide YY (PYY), both involved in the regulation of appetite, body weight, and glucose metabolism [29]. Consequently, we found that the endogenous production of GLP-1 was increased (i.e., higher mRNA expression, peptide production and secretion, as well as an increase in the number of producing cells). Hence, the healthier phenotype was linked to greater GLP-1 and PYY levels in the portal vein blood [25–27]. Later, this finding was reproduced using resistant starches or arabinoxylans [30–33]. The molecular event explaining how fermentation increases gut peptides is generally attributed to the capacity of SCFAs to interact with specific G protein-coupled receptors such as GPR-43 (also referred to as FFAR2 for free fatty acid receptor 2) and GPR-41 (also referred to as FFAR3) [34–36] (Fig. 1). Mice with deletion of these specific receptors are resistant to the beneficial effects of exposure to SCFAs or specific prebiotics and are characterized by an altered production of GLP-1 and PYY [37, 38, 39•]. Notably, in humans, we and others reported that modulation of the microbiome using prebiotics not only increased satiety and reduced hunger and appetite, but also increased the production of GLP-1 and PYY levels in both lean and obese volunteers [40–50] (for review [51]).

In 2018, a very interesting study was published by Liping Zhao and colleagues wherein they conducted a randomized clinical study that compared the impact of two isoenergetic diets that differed in their concentrations of prebiotics on the modulation of the microbiome [52••]. In this study, they performed an in-depth investigation of the mechanisms potentially explaining the beneficial effects of a diet rich in fibers; hence, they analyzed the composition of the gut microbiome (i.e., taxa and gene functions), the levels of plasma GLP-1 and PYY, and the measurements of SCFAs and other metabolites such as hydrogen sulfur and indoles. All subjects enrolled were diagnosed with type 2 diabetes and were treated with acarbose. At the completion of the study (i.e., 84 days), both groups exhibited a significantly lower level of hemoglobin A1c (HbA1c); however, a greater reduction was observed in the group that received the high-fiber diet (composed of whole grains, traditional Chinese medicinal foods, and prebiotics) than in the control group, which received the usual care and was administered a diet that followed the normal dietary recommendations of the Chinese Diabetes Society. Only 50% of subjects in the control group achieved adequate glycemic control (i.e., an HbA1c level < 7%), whereas this goal was reached by 89% of the patients consuming the high-fiber diet. Similarly, the experimental group achieved greater reductions in body weight (4.20 ± 0.93% of body weight loss versus 1.45 ± 0.91% in the control group).

Since the microbial and metabolic profiles of both groups did not differ at the beginning of the study, the modulation of the gut microbiome by the fibers was likely to explain the phenotype. To causally link the different microbial and metabolic profile to the observed phenotype, the authors transplanted the gut microbiota from the participants into germ-free mice. Interestingly, when the mice were colonized with the gut microbiota collected after the dietary interventions, they displayed different metabolic improvements than mice that were colonized with the microbiota harvested before the intervention. However, the most interesting finding was that the mice colonized with the gut microbiota from the patients treated with the experimental diet also showed lower fasting and postprandial blood glucose levels than the control mice. It is worth noting that the beneficial effects were likely due to the combination of the different fermentable fibers and plants contained in the diet. Therefore, the synergistic effects observed are probably resulting from the impact on the microbiota of both, the sources of non-digestible carbohydrates and the different polyphenolic compounds (discussed in the next chapter of this review). These observations led the authors to conclude that the changes in the microbiome induced by increasing the consumption of non-digestible carbohydrates were sufficient to improve the metabolic parameters of patients with type 2 diabetes. Among the mechanisms, here again the levels of SCFAs correlated with improvements in several metabolic parameters, such as lower glycemia, blood lipid levels, and fat mass, as well as increased blood levels of GLP-1 and PYY [52••].

Although this study and other related studies have correlated the abundance of SCFAs and gut peptides with metabolic effects, researchers have not yet been able to completely ascertain whether the duo of SCFAs and gut hormones completely explain the observed results in humans.

### Polyphenols

#### Polyphenols and Health

The first definition of prebiotics—a non-digestible food ingredient that beneficially affects the host by selectively stimulating the growth and/or activity of one or a limited number of bacteria in the colon, and thus improves host health [53]—was tailored to fibers. However, based on a fast-growing body of literature, this definition has continued to evolve over time [54], with the latest consensus being that a prebiotic is a substrate that is selectively utilized by host microorganisms conferring a health benefit, thus expanding the concept to also include non-carbohydrate substances [55••]. Indeed, some beneficial microbes also gain proliferating advantages from other compounds that are not covered by the initial description of a prebiotic. Polyphenols fall into this category.

Polyphenols constitute a large heterogenous family of over 8000 phytochemicals characterized by hydroxylated phenyl moieties [56]. They are classified based on their chemical structure and complexity (i.e., the number of phenolic rings and substituting groups), but generally they are broken down into two categories: flavonoids and non-flavonoids [57]. Non-flavonoids include phenolic acids, stilbenes, and lignans. Flavonoids are further divided into subclasses including flavanones, flavones, dihydroflavonols, flavonols, flavanols, anthocyanidins, isoflavones, and proanthocyanidins. Polyphenols are bioactive compounds that are second only to carbohydrates when it comes to abundancy in plants, where they have a protective role against, for example, damage from UV radiation and pathogens.

Although they have been used in traditional medicine for a long time, we are only now beginning to understand their mechanisms of action and accumulating literature is demonstrating that the consumption of polyphenol rich foods and beverages such as tea, coffee, fruits, vegetables, cereals, and wine could benefit human health. It is suggested that various polyphenols possess properties that could have preventive and/or therapeutic effects for cardiovascular diseases, neurodegenerative disorders, cancer, allergies, diabetes, and obesity [58–60].

#### Polyphenols and Obesity

There are several in vivo studies, both in animals and in humans that suggest a negative association between polyphenol intake and body weight [58, 60, 61•, 62–64, 65•, 66]. Perhaps the best-studied polyphenols in this context are the polyphenols from green tea [67, 68]. Green tea is the dried leaves of *Camellia sinensis* plants. It contains the highest amounts of phenolic compounds and exhibits the highest antioxidant capabilities of any major tea type. It is rich in flavonoids (e.g., catechin, epicatechin, epicatechin gallate, epigallocatechin gallate, and proanthocyanidins). Of these, epigallocatechin gallate (EGCG) is considered the most significant active component. EGCG has been attributed antioxidant, anti-inflammatory and anti-mutagenic properties and is thought to help prevent weight gain [63, 67–70].

Besides (green) tea, many other sources of polyphenols have been studied for their potential preventive and therapeutic properties [63, 65•]. Coffee is rich in 3-caffeoylquinic acid, fruits such as blueberries have anthocyanins, red grapes and wine are a source of resveratrol, and spice like turmeric contains curcumin. All of these foods have been inversely associated with the metabolic syndrome and BMI in epidemiological studies [60].

It is important to note that some human epidemiological studies have shown inconsistent results. This may indicate that the benefits of polyphenols may depend on the method of administration or on the population studied [63, 65•]. Also, the number of interventional studies providing direct evidence for these associations are still few, use relatively low doses of specific polyphenols, and occur over a short period of time [65•].

#### Mechanisms of Action of Polyphenols

How polyphenols exert their beneficial effects in the context of obesity is still a matter of debate. Polyphenols possess electron-donating phenolic groups in their structures, which allow them to locally prevent cellular damage caused by reactive oxygen species in the intestinal tract. However, although their antioxidant capacity is often cited, recent research suggests this is not the major cause for their effects on body fat accumulation [67]. A number of polyphenols have been shown to reduce nutrient intake in the gastrointestinal tract by interacting with and inhibiting digestive enzymes, thereby hampering starch, lipid, and protein digestion and absorption to reduce energy efficiency [71–76]. Other potential mechanisms of action include reduction of inflammation, modulation of glucose homeostasis, suppression of adipogenesis and lipid synthesis, increase of energy expenditure via thermogenesis, stimulation of fat oxidation, and excretion of fecal lipids [60, 61•, 62, 77, 78]. The precise underlying molecular mechanisms, however, remain unclear. One possibility, the so-called “AMPK hypothesis” proposed by Yang et al., is that the polyphenols activate AMPK (adenosine monophosphate-activated protein kinase) [61•, 79]. This “master switch” is ubiquitously expressed and serves as the master metabolic molecule that regulates how cells process energy, making it a heavily pursued target for treatment of metabolic diseases [80, 81]. Polyphenols have been shown in vivo and in vitro to activate AMPK.

#### Polyphenols and Microbiota

Most polyphenols, however, display poor bioavailability. They are not well absorbed in the small intestine [82] and are rapidly metabolized and excreted in urine and feces. However, when reaching the colon, they come in direct contact with the gut microbes, resulting in a complex and multidirectional interaction.

First, the antioxidant capacity of polyphenols ensures the protection of the colonic epithelial and mucosa architecture and shapes the environment in which gut microbes can thrive [83]. Second, since the vast majority of the polyphenols reach the colon almost unaltered, they are able to modify the intestinal microbiota, possibly affecting its capacity for energy harvest, and contributing to the improvement of health [84, 85]. As a major source of nutrients for some microbes residing in the colon, polyphenols may also act as prebiotics and favor the growth of specific species [83, 86–91]. In addition, polyphenols may be involved in the adhesion of probiotic bacteria to the intestinal epithelia [92, 93]. This could be important for their colonization and for their subsequent efficacy to exert physiological effects [94].

Third, polyphenols are subjected to catabolism by the microbiota and are degraded into low molecular weight bioactive compounds that are more easily absorbed through the intestinal barrier and are capable of exerting effects in peripheric organs [86, 95–98]. Perhaps the best known metabolites are the phenolic acids, to which several potential health benefits have been attributed, such as anti-microbial activities, anti-inflammatory and anti-oxidative properties, reinforcement of the gut barrier, and also for regulating cyclooxygenase 2 activity [99•, 100]. Interestingly, it has been suggested that polyphenols may also be able to induce the production of SCFAs by colonic bacteria [67, 101–104]. This occurs either by the growth promotion of SCFA-producing bacteria (prebiotic effect), or by stimulation of their metabolic functions [105]. Finally, the intrinsic toxicity of the phenolic group gives polyphenols antibacterial activity [106–108]. As this may prevent biofilm formation and favor some bacterial species above others this feature allows polyphenols to further impact the microbial composition [109].

Although short-term animal studies clearly showed an effect of polyphenols on gut microbiota composition and obesity [110–114], it remains to be established whether these changes are significant enough to explain, at least in part, the health benefits of polyphenols in the long-term in human populations. For example, it has been described how the long-term supplementation of green tea did not change the gut microbiota in humans [115].

#### Polyphenols and *Akkermansia muciniphila*

*Akkermansia muciniphila* is a Gram-negative mucin-degrading bacterium that, in contrast to most other gut microbes, resides within the mucus layer that covers the gut. It makes up 1–5% of the microbes in a healthy human gut, but its abundance is strongly reduced in the intestine of obese individuals. *A. muciniphila* is inversely associated with obesity, diabetes, cardiometabolic diseases, and low-grade inflammation, and the supplementation with *A. muciniphila* has been linked to beneficial effects in a variety of preclinical models [116–118].

In 2014, a study demonstrated that a polyphenol-rich cranberry extract prevented weight gain and ameliorated several features of the metabolic syndrome in diet-induced obese mice [111]. This was associated with a strong increase in the abundance of *A. muciniphila* in the gut microbiota. The exact mechanisms behind this increase are not known, but interestingly, it was shown that cranberry proanthocyanidins are also associated with an increase in the production of mucus, a major energy source for these mucin-degrading bacteria [119]. The increase in *Akkermansia* was later also observed in experiments that used extracts from grapes, that, like berries, contain abundant proanthocyanidins [85, 120], and also in studies using other types of berries and polyphenols [84, 121–124]. This suggests that polyphenols may exert at least part of their beneficial effects via a prebiotic effect on certain bacteria such as *Akkermansia*.

#### Polyphenols as an Anti-obesity Drug

The amplitude of the anti-obesity effects in humans will probably depend on many factors. Beside the classical interindividual variations (genetics and differences in gut microbiota composition), other factors such as how and when polyphenols are administered, may play a role. Since polyphenols and fibers share common plant-based sources, they are often consumed together. Interactions between polyphenols and polysaccharides are therefore inevitable. These interactions can have profound consequences on the extractability, bioavailability, functional characteristics, and on the fermentation kinetics of dietary fibers and/or polyphenols [125]. A study by Edwards et al. [126] aims at exploring this interplay and may lead to new product designs that further optimize the positive actions of dietary fibers and polyphenols. For example, because fibers are fermented, they may selectively increase the activity of specific bacteria which in turn may potentialize the presence of bacteria responsible for polyphenol catabolism. Moreover, polyphenols may also influence the fermentation of the fibers as numerous polyphenols exhibit both anti-microbial and prebiotic effects. Therefore, understanding the complex interaction between both dietary fibers and polyphenolic compounds will pave the way for designing future nutritional composition or food combination with an optimal health benefit [126].

Another critical factor is dosage. It is important to achieve high enough levels to have an effect, but overconsumption of polyphenols may not be without risk and caution is advised when preparing concentrated extracts [127, 128].

The anti-obesity effects of polyphenols may be further enhanced by habitual exercise [129, 130]. In vitro data suggest that catechins inhibit the enzyme catechol-O-methyltransferase (COMT). This enzyme is partially responsible for the breakdown of norepinephrine, which is involved in the mobilization of fat from adipose tissues. If the lipids that are liberated are not maximally utilized by increasing physical activity, they will be stored again, attenuating the anti-obesity effects; however, whether this mechanism may explain the in vivo effects remains controversial [131].

## Conclusion

While the obesity problem is often reduced to a simple mathematical imbalance between energy intake and energy expenditure, understanding how it develops and progresses is a complex problem that requires comprehension of multiple genetic, biologic, environmental, and socioeconomic factors. Countering its advance will require multidisciplinary and innovative approaches, combining dietary and behavioral changes with nutritional or therapeutic supplements, if needed. One of the components that can directly influence the outcome of such strategies is the gut microbiota. Indeed, the microbial community that colonizes the border between lumen of the gut and the inside of the body plays a decisive role in the host metabolism.

Non-digestible carbohydrates and polyphenols, two types of substrates that are metabolized by the gut microbes, represent an interesting set of tools to bend the microbiota to our advantage because they can modify the gut microbiota composition and be converted by the microbiota into useful metabolites. Which fibers or polyphenols will prove the most suited to maximize beneficial effects will probably depend on the specific situation and the individual (and its microbiota). While we have gained a lot of insights regarding the effects and mechanism of actions of these promising compounds, further investigations are still warranted in order to design optimal associations of compounds according to the health benefit expected, that is improving weight management, as well as glucose metabolism or even anti-inflammatory properties.
